# Efficacy of idelalisib and rituximab in relapsed/refractory chronic lymphocytic leukemia treated outside of clinical trials. A report of the Gimema Working Group

**DOI:** 10.1002/hon.2861

**Published:** 2021-03-26

**Authors:** Gian Matteo Rigolin, Francesco Cavazzini, Alfonso Piciocchi, Valentina Arena, Andrea Visentin, Gianluigi Reda, Giulia Zamprogna, Francesca Cibien, Orsola Vitagliano, Marta Coscia, Lucia Farina, Gianluca Gaidano, Roberta Murru, Marzia Varettoni, Rossella Paolini, Paolo Sportoletti, Daniela Pietrasanta, Anna Lia Molinari, Francesca M. Quaglia, Luca Laurenti, Roberto Marasca, Monia Marchetti, Francesca R. Mauro, Enrico Crea, Marco Vignetti, Massimo Gentile, Marco Montillo, Robin Foà, Antonio Cuneo, GIMEMA Group

**Affiliations:** ^1^ Department of Medical Sciences Hematology Section University of Ferrara Cona ‐ Ferrara Italy; ^2^ GIMEMA Foundation Rome Italy; ^3^ Department of Medicine Hematology and Clinical Immunology Unit University of Padua Padua Italy; ^4^ Ematologia, Fondazione IRCCS Ca’ Granda Ospedale Maggiore Policlinico di Milano Milan Italy; ^5^ Hematology Niguarda Cancer Centre ASST Grande Ospedale Metropolitano Niguarda Milan Italy; ^6^ Hematology, Ospedale Ca’ Foncello Treviso Italy; ^7^ Hematology Cardarelli Hospital Naples Italy; ^8^ Department of Molecular Biotechnology and Health Sciences University of Torino Turin Italy; ^9^ Division of Hematology A.O.U. Città della Salute e della Scienza di Torino Turin Italy; ^10^ Division of Hematology Fondazione IRCCS Istituto Nazionale dei Tumori, Milano Milano Italy; ^11^ Department of Translational Medicine Division of Hematology Università del Piemonte Orientale Novara Italy; ^12^ Hematology and Stem Cell Transplantation Unit Ospedale Oncologico A. Businco ARNAS “G. Brotzu” Cagliari Italy; ^13^ Division of Hematology Fondazione IRCCS Policlinico San Matteo Pavia Italy; ^14^ Hematology Unit Rovigo General Hospital Rovigo Italy; ^15^ Department of Medicine and Surgery Institute of Hematology and Centre for Hemato‐Oncological Research Ospedale S. Maria della Misericordia Perugia Italy; ^16^ Hematology Division Dipartimento Internistico Struttura Complessa di Ematologia Ospedale civile SS Antonio e Biagio Alessandria Italy; ^17^ Hematology Ospedale degli Infermi Rimini Italy; ^18^ Department of Medicine Section of Hematology University of Verona Verona Italy; ^19^ Fondazione Policlinico Universitario A Gemelli. Roma IRCCS Rome Italy; ^20^ Department of Medical and Surgical Sciences Section of Hematology University of Modena and Reggio Emilia Modena Italy; ^21^ Oncology Unit Cardinal Massaia Hospital Asti Italy; ^22^ Department of Translational and Precision Medicine Hematology ‘Sapienza’ University Rome Italy; ^23^ Department of Onco‐Hematology Hematology Unit A.O. of Cosenza Cosenza Italy

**Keywords:** chronic lymphocytic leukemia, idelalisib, real‐world evidence

## Abstract

Because the efficacy of new drugs reported in trials may not translate into similar results when used in the real‐life, we analyzed the efficacy of idelalisib and rituximab (IR) in 149 patients with relapsed/refractory chronic lymphocytic leukemia treated at 34 GIMEMA centers. Median progression‐free survival (PFS) and overall survival were 22.9 and 44.5 months, respectively; performance status (PS) ≥2 and ≥3 previous lines of therapy were associated with shorter PFS and overall survival (OS). 48% of patients were on treatment at 12 months; the experience of the centers (≥5 treated patients) and PS 0–1 were associated with a significantly longer treatment duration (*p* = 0.015 and *p* = 0.002, respectively). TP53 disruption had no prognostic significance. The overall response rate to subsequent treatment was 49.2%, with median OS of 15.5 months and not reached in patients who discontinued, respectively, for progression and for toxicity (*p* < 0.01). Treatment breaks ≥14 days were recorded in 96% of patients and adverse events mirrored those reported in trials. In conclusion, this real‐life analysis showed that IR treatment duration was longer at experienced centers, that the ECOG PS and ≥3 lines of previous therapy are strong prognostic factor and that the overall outcome with this regimen was superimposable to that reported in a randomized trial.

## 1 INTRODUCTION

The introduction of kinase targeted treatment has represented a major advance in the management of patients with relapsed refractory (R/R) chronic lymphocytic leukemia (CLL), including patients with genetically defined high‐risk disease and fludarabine‐refractory disease.^1^, ^2^, ^3^ The majority of R/R patients can be effectively treated with the Bruton tyrosine kinase (BTK) inhibitor ibrutinib, which has been associated with a 71% overall response rate (ORR) and a 75% estimated progression‐free survival (PFS) rate at 26 months, with a 7‐year PFS of 34%.^4^, ^5^

The selective PI3Kdelta inhibitor idelalisib given as single agent produced objective responses in 81% of patients who had received a median of five previous lines of treatment.^6^ The combination of idelalisib and rituximab (IR) in a Phase 3 trial including patients who were deemed ineligible to further chemoimmunotherapy showed a 93% PFS rate at 24 weeks,^7^ a median PFS of 20.3 months and a median overall survival (OS) of 40.6 months.^8^ In another Phase 3 trial, a median PFS of 15.8 months was reported with IR^9^ and an increased incidence of adverse events leading to treatment interruptions and/or discontinuation in the majority of patients was noted.^8^, ^9^ Therefore, expert opinions for the management of adverse events have been proposed to improve adherence to this regimen.^10^, ^11^

The efficacy of new drugs reported in clinical trials may not translate into similar results when used in the day‐to‐day real‐life practice.^12^, ^13^ This prompted us to carry out an observational retrospective‐prospective study on the long‐term efficacy and safety of IR in R/R CLL patients treated outside of clinical trials in 34 centers of the Gruppo Italiano Malattie EMatologiche dell’Adulto (GIMEMA) cooperative study group.

Accepting the limitations of observational studies, we included, in addition to PFS, the most objective endpoints for this analysis, i.e., the percentage of patients on treatment at different time points and OS, and also investigated how baseline clinical and biologic features could impact on the outcome of treatment and analyzed outcome after idelalisib discontinuation.

## 2 MATERIALS AND METHODS

### 2.1 Patients

Patients treated with IR between 2014 and 2017 at GIMEMA centers were selected for this analysis from local pharmacy databases and/or from unit‐specific databases. The inclusion criteria in this observational retrospective study were (i) diagnosis of CLL according to the National Cancer Institute (NCI) recommendations,^14^ (ii) age ≥18 years, (iii) one previous treatment with alkylating agents and/or purine analogues with or without monoclonal antibodies, (iv) progression requiring therapy,^14^ (v) treatment with at least one dose of IR. Patients were excluded if they had a Richter's syndrome transformation, HIV infection, active HCV, or HBV infection. The study was registered at ClinicalTrials.gov (NCT03545035) and was approved by the local Ethics Committees.

### 2.2 Study design and endpoints

Data were obtained from the medical files and entered into case record forms by the treating physicians. Computerized and manual consistency checks were performed by the data managers of the GIMEMA Data Center. Treatment response and disease progression were assessed according to the NCI criteria.^14^ The primary endpoint was PFS at 12 months from the start of treatment. Subjects who withdrew from the study without progression were censored at the date of the last assessment. Subjects without post‐baseline assessments but known to be alive were censored at the time of the first dose of the study drug. Secondary endpoints were (i) the ORR, assessed in all patients who started treatment, (ii) OS calculated from the date of the first dose of the study drug up to the date of death from any cause, and (iii) the percentage of patients on treatment at 12 months. Patients without follow‐up assessment were censored at the day of the last treatment administration. Being aware of the difficulty in obtaining a detailed description of adverse events (AEs) in a retrospective analysis, we asked clinicians to report any clinically significant AE deemed possibly related to idelalisib and/or rituximab according to the NCI Common Terminology Criteria for AE version 4.0.

### 2.3 Statistical analysis

Patients characteristics were summarized by means of cross‐tabulations for categorical variables or by means of median and range for continuous variables. Nonparametric tests were performed for comparisons between groups (Chi‐Squared and Fisher Exact test in case of categorical variables or response rate, Wilcoxon and Kruskal–Wallis test in case of continuous variables) and logistic regression was applied in multivariate analysis. Survival distributions were estimated using the Kaplan–Meier Product Limit estimator. Subgroups comparisons were performed for descriptive purposes and differences were evaluated by means of Log‐Rank test in univariate analysis and by means of the Cox regression model in multivariate analysis. Confidence intervals were estimated at the 95% level and all tests were two‐sided, accepting *p* < 0.05 as indicative of a statistically significant difference. All analyses were performed using the SAS system software (version 9.4) and R statistical software.

## 3 RESULTS

### 3.1 Patients

One‐hundred and forty‐nine R/R CLL patients from 34 Italian centers were included in the study (Table S1). Twelve centers reported data on at least five patients (69.8% of the total population) and the remaining 22 centers reported less than five patients (30.2% of the total patient population). Forty‐five percent of cases had received three or more previous lines of therapy, previous exposure to ibrutinib occurred in 12.9% of the patients.

The baseline characteristics are shown in Table 1. Median age was 71.4 years (range 46–90) and 55.7% of cases were >70 years. Two or more comorbidities were present in 56.1% of patients; 58.7%, 30%, 8%, 3.3% of them had an ECOG performance status (PS) of 0, 1, 2, and 3, respectively; 54.6% had a creatinine clearance ≤70 ml/min; 48.3% had an advanced disease stage (i.e., Rai III‐IV or Binet C). Seventy‐two percent had an unmutated configuration of the immunoglobulin genes (U‐IGHV) (data available in 100 out of 149 patients) and 34.9% carried a *TP53* disruption (i.e., mutated *TP53* and/or 17p13 deletion).

**TABLE 1:** Baseline characteristics

Variable	Idelalisib‐Rituximab *n* = 149 (%)
Age (median [range]) years	71.4 (46–90)
Age ≤70/>70 years	66 (44.3)/83 (55.7)
Gender M/F	100 (67.1)/49 (32.9)
ECOG PS (%) 0–1/≥2	125 (88.7)/16 (11.3)
Comorbidities 0–1/≥2	43 (43.9)/55 (56.1)
Creatinine clearance (ml/min) ≤70/>70	65 (54.6)/54 (45.4)
Stage Rai III/IV or Binet C no/yes	77 (51.7)/72 (48.3)
Bulky lymph nodes (>5 cm) no/yes	91 (82.0)/20 (18.0)
*TP53* disruption yes/no^a^	52 (34.9)/97 (65.1)
*IGHV* mutated/unmutated	28 (28)/72 (72.0)
Previous lines of therapy <3/≥3	82 (55.0)/67 (45.0)

Abbreviations: F, female; M, male.

^a^ Del17p and/or *TP53* mut.

### 3.2 Treatment with IR

**FIGURE 1:**
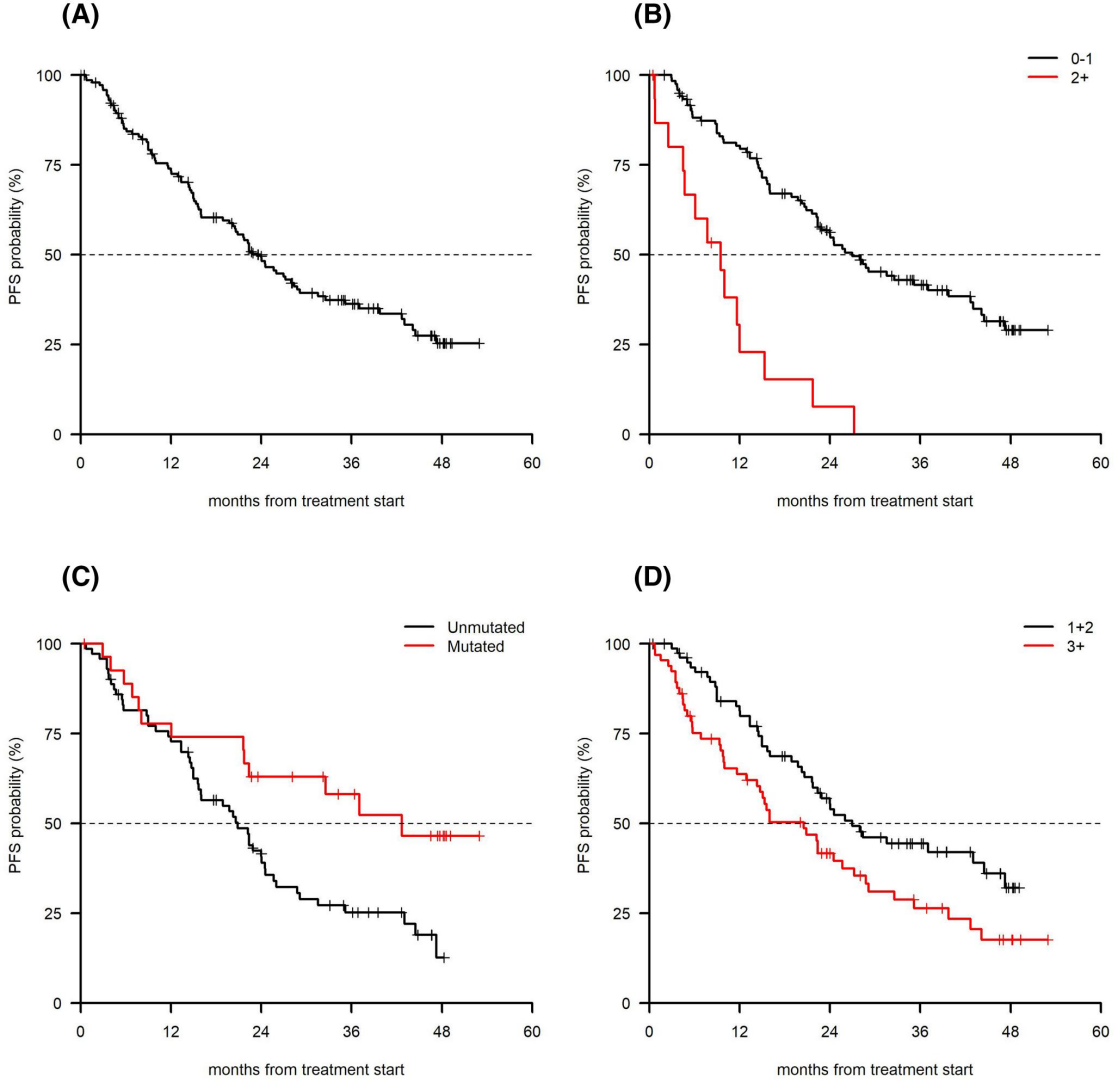
(A) Overall PFS, (B) PFS by ECOG ( *p* < 0.001), (C) PFS by IGHV status (*p* = 0.01), and (D) PFS by number of previous lines of therapy (*p* = 0.02). PFS, progression‐free survival

At a median follow‐up of 39.4 months, the median PFS was 22.9 months (95% CI 20.5–28.8, Figure 1A). Factors predicting for a shorter PFS at univariate analysis (Table 2) were an ECOG PS ≥ 2 (median 9.5 months [95% CI 4.7–21.7] vs. 26.9 months [95% CI 22.4–39.8] *p* < 0.001), Figure 1B), U‐IGHV (median 20.9 months [95% CI 15.7–24.5] vs. 42.7 months [95% CI 22.4–NA] *p* = 0.01; (Figure 1C), and ≥3 lines of therapy (median 20.5 [95% CI 14.3–28.8] vs. 27.0 months [95% CI 21.7–47.3] *p* = 0.02; Figure 1D), with a borderline significance (*p* = 0.06) for advanced stage. A shorter PFS was noted in the 22 centers that included <5 patients (median 22.4 [95% CI 15.1–28]), compared to the 12 centers that enrolled ≥5 patients (median 24.5 [95% CI 20.6–42.7], with a borderline statistical significance; *p* = 0.06). *TP53* disruption had no impact on PFS, as was the case with age (cut‐off 70 years), creatinine clearance [cut‐off 70 ml/minute (min)] and the presence or absence of two or more comorbidities. PS ≥ 2 (*p* < 0.001), U‐IGHV (*p* = 0.006) and ≥3 lines of therapy (*p* = 0.02) were associated with a shorter PFS at multivariate analysis (Table 2).

**TABLE 2:** Progression‐free survival: Univariate and multivariate analysis

Variable	Univariate analysis		Multivariate analysis	
	HR (95% CI)	*p*	HR (95% CI)	*p*
Age (median) years	0.99 (0.96–1.01)	0.27	‐	‐
Age ≤70/>70 years	1.16 (0.76–1.76)	0.50	‐	‐
Gender M/F	0.98 (0.63–1.53)	0.94	‐	‐
ECOG PS 0–1/≥2	0.21 (0.11–0.38)	<0.001	0.19 (0.06–0.34)	<0.001
Comorbidities 0–1/≥2	0.93 (0.56–1.54)	0.79	‐	‐
Creatinine clearance (ml/min) ≤70/>70	0.73 (0.47–1.13)	0.15	‐	‐
Stage Rai III/IV or Binet C yes/no	1.50 (0.99–2.27)	0.06	‐	‐
Bulky lymph nodes (>5 cm) no/yes	0.65 (0.37–1.13)	0.12	‐	‐
*TP53* disruption yes/no^a^	0.97 (0.63–1.49)	0.89	‐	‐
*IGHV* mutated/unmutated	0.46 (0.25–0.84)	0.01	0.40 (0.21–0.77)	0.006
Previous lines of therapy <3/≥3	0.61 (0.40–0.92)	0.02	0.56 (0.34–0.93)	0.02
No. patients per center ≥5 versus <5	0.66 (0.42–1.02)	0.06	‐	‐

^a^ Del17p and/or *TP53* mut.

Because it was not possible to document response according to the NCI criteria due to the heterogeneity in response assessment across centers, we recorded as response what each treating clinician graded as partial or complete remission (PR, CR). The ORR (CR + PR) was 72.5%; age ≤70 years (*p* = 0.04) and a PS < 2 (*p* = 0.02) were associated with a higher ORR (Table S2).

**FIGURE 2:**
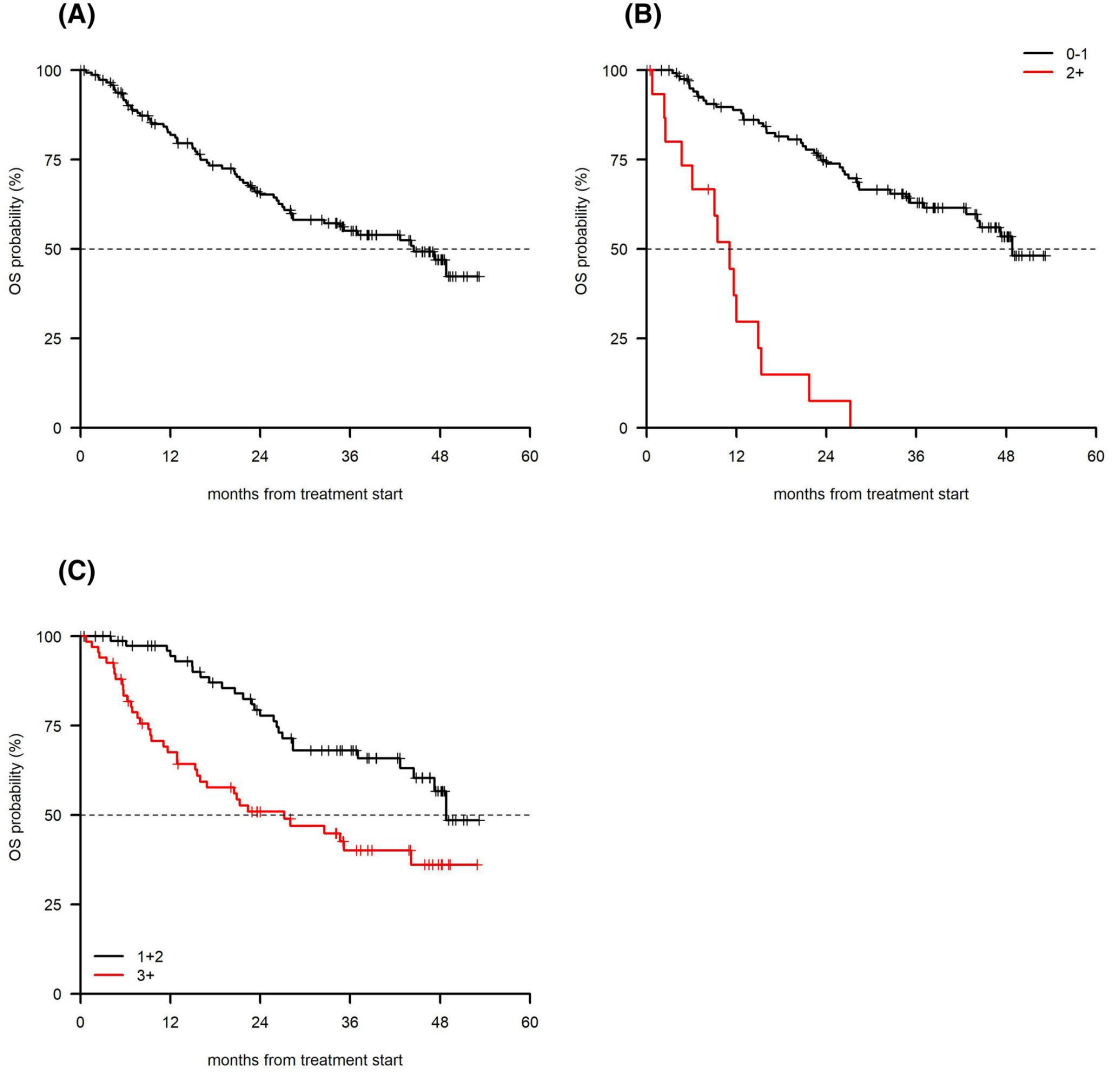
(A) Overall OS, (B) OS by PS (*p* < 0.001), and (C) OS by number of previous line of therapy (*p* = 0.001). OS, overall survival; PS, performance status

The median OS was 44.5 months (95% CI 32.5–NA; Figure 2A). In univariate analysis, a shorter OS was associated with a PS ≥ 2 (median 11.1 months [95% CI 6.12–21.7] vs. 48.8 months [95% CI 44.14–NA] *p* < 0.001, Figure 2B), advanced stage (median 35.2 months [95% CI 20.6–NA] vs. not reached [95% CI 42.7 NA] *p* = 0.02), bulky disease (median 25.2 months [95% CI 11.6–NA] vs. not reached *p* = 0.01) and ≥3 lines of therapy (median 27.2 months [95% CI 16.0‐NA] vs. 48.8 months [95% CI 44.5‐NA], *p* = 0.001; Figure 2C) with a borderline significance for U‐IGHV (*p* = 0.07). *TP53* disruption and the size of the centers had no impact on OS. PS ≥ 2 (*p* < 0.001) and ≥3 lines of therapy (*p* = 0.046) were associated with a shorter OS at multivariate analysis (Table 3).

**TABLE 3:** OS Univariate and multivariate analysis

Variable	Univariate analysis		Multivariate analysis	
	HR (95% CI)	*p*	HR (95% CI)	*p*
Age (median) years	1.01 (0.98–1.04)	0.51	‐	‐
Age ≤70/>70 years	0.97 (0.59–1.60)	0.90	‐	‐
Gender M/F	0.86 (0.51–1.44)	0.57	‐	‐
ECOG PS (%) 0–1/≥2	0.12 (0.06–0.22)	<0.001	0.14 (0.07–0.30)	<0.001
Comorbidities 0–1/≥2	0.81 (0.43–1.50)	0.49	‐	‐
Creatinine clearance (ml/min) ≤70/>70	1.08 (0.64–1.84)	0.77	‐	‐
Stage Rai III/IV or Binet C yes/no	1.83 (1.11–3.01)	0.02	‐	‐
Bulky lymph nodes (>5 cm) no/yes	0.46 (0.25–0.84)	0.01	‐	‐
*TP53* disruption yes/no^a^	0.93 (0.56–1.54)	0.77	‐	‐
*IGHV* mutated/unmutated	0.51 (0.24–1.06)	0.07	‐	‐
Previous lines of therapy <3/≥3	0.44 (0.26–0.72)	0.001	0.54 (0.30–0.99)	0.046
No. patients per center ≥5 versus <5	0.82 (0.48–1.40)	0.47	‐	‐

Abbreviations: OS, overall survival; PS, performance status.

^a^ Del17p and/or *TP53* mut.

Forty‐eight percent, 24.3%, and 11.8% of patients were still receiving the study drug at 12, 24, and 36 months, respectively. Discontinuation within 12 months was due to toxicity in 60.8% of cases. Only 31.1% of the patients were on treatment at 12 months in centers reporting <5 patients versus 55.8% on treatment in centers that included ≥5 patients (*p* = 0.007). Descriptive data of patients in relation to the treatment duration (≥12 months vs. < 12) are reported in (Table S3).

A significantly shorter treatment duration (<12 months) was recorded in patients with advanced clinical stage, with *TP53* disruption (*p* < 0.048), with PS ≥ 2 (*p* = <0.004) and in centers that included <5 patients (*p* = 0.003), the latter two parameters being significant at multivariate analysis (Table 4).

**TABLE 4:** Univariate and multivariate analysis affecting idelalisib‐rituximab treatment time ≥12 versus <12 months

	Univariate analysis		Multivariate analysis	
Variable	OR (95%CI)	*p*	OR (95%CI)	*p*
Age (median) years	1.02 (0.98–1.06)	0.34	‐	‐
Age ≤70/>70 years	0.66 (0.34–1.29)	0.22	‐	‐
Gender M/F	0.71 (0.35–1.44)	0.35	‐	‐
ECOG PS (%) ≥2/0–1	0.05 (0.01–0.37)	0.004	0.04 (0.05–0.31)	0.002
Comorbidities 0–1/≥2	0.47 (0.21–1.05)	0.07	‐	‐
Creatinine clearance (ml/min) ≤70/>70	0.93 (0.45–1.94)	0.85	‐	‐
Stage Rai III/IV or Binet C yes/no	0.45 (0.23–0.88)	0.02	‐	‐
Bulky lymph nodes (>5 cm) no/yes	0.90 (0.33–2.42)	0.84	‐	‐
*TP53* disruption yes/no^a^	2.03 (1.01–4.07)	0.048	‐	‐
*IGHV* mutated/unmutated	0.87 (0.36–2.08)	0.75	‐	‐
Previous lines of therapy <3/≥3	1.79 (0.92–3.48)	0.09	‐	‐
No. patients per center <5 versus ≥5	2.727 (1.29–5.75)	0.008	2.74 (1.21–6.20)	0.015

^a^ Del17p and/or *TP53* mut.

**FIGURE 3:**
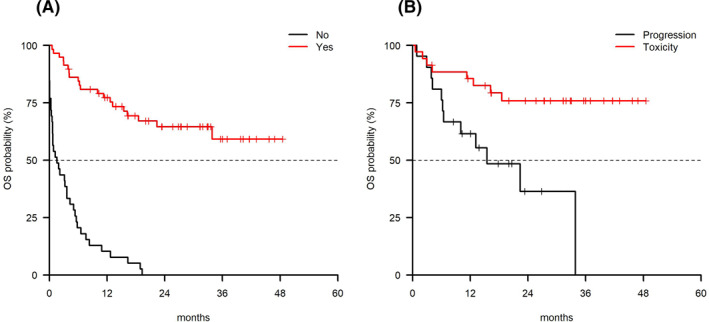
(A) OS by subsequent treatment (*p* < 0.0001) and (B) reason for discontinuation (*p* < 0.01). OS, overall survival

Fifty‐nine patients received a subsequent treatment with an ORR of 49.2% and a median OS not reached at a median follow‐up of 11 months (range 0.1–45.6). The only predictor for response to the new treatment was having received <2 lines of therapy prior to IR (ORR 11/14 = 78.6% vs. 18/45 = 40.0% for patients with <2 and ≥2 previous lines of therapy respectively, *p* = 0.02). A significantly shorter OS was observed in patients who did not receive further treatment in comparison with those who received a subsequent treatment (1.6 months [95% CI 0.7–4.3] vs. NR [95% CI 33.8–NA] respectively, *p* < 0.0001, Figure 3A). Among patients who received a subsequent treatment, the OS was better when discontinuation was due to adverse events versus progression (median OS not reached vs. 15.5 months [95% CI 6.48–NA] *p* < 0.01; Figure 3B).

### 3.3 Safety

A detailed report of grade 3–5 AE in 117 patients is shown in Table S4. AE grade ≥3 during IR treatment were neutropenia in 33.3% of patients, gastrointestinal disorders, including diarrhea and colitis in 27.3%, infections in 29.7%, transaminitis in 6%, and rash in 6.8%. Two fatal events (one pneumonitis and one leukoencephalopathy) were observed (0.1%). Temporary treatment breaks of ≥14 days were recorded in 143 patients (96%) and dose reductions in 58 patients (38.9%).

## 4 DISCUSSION

To minimize selection and attrition biases as well as imprecise reporting of data inherent to observational studies,^15^, ^16^, ^17^, ^18^ we encouraged clinicians to report all patients who initiated IR treatment, we analyzed the reported data according to the intention‐to‐treat principle, we updated the outcome in all patients, and we performed computerized and manual consistency checks on each case report form. Furthermore, we included, besides PFS, objective efficacy measures of the IR regimen, such as the percentage of patients on treatment at 12 months and OS.

Idelalisib containing regimens have been investigated in clinical trials that have shown a higher ORR and better PFS compared to anti‐CD20 monoclonal antibodies in R/R CLL, including patients with high‐risk disease.^8^, ^19^ However, in light of drug‐specific AE, more patients discontinued IR treatment due to toxicity than because of disease progression or death. This has raised the question on how much this potentially efficacious regimen has been utilized in the real‐life clinical practice without the constraints represented by rigorous inclusion criteria and selection of centers accustomed to clinical trials.

To answer this question, we carried out an analysis based on a relatively large dataset of CLL patients treated off‐trial with IR and followed for a prolonged period of time in 34 centers of the Italian GIMEMA cooperative group. Our study provides therefore a real‐life evaluation of data reflecting a widespread utilization of IR in our country, with a majority of the investigators reporting less than five treated patients. We were able to analyze the efficacy and tolerability of this combination with a minimum follow‐up for living patients of 12 months and to assess the outcome after IR discontinuation.

The baseline characteristics of the patients included in this analysis are similar to those of the Phase 3 trial comparing IR with placebo and rituximab,^7^, ^8^ with a median age of 71 years, the majority of patients in advanced stage, reduced renal function, multiple previous lines of treatment, adverse genetic characteristics, and symptoms affecting the PS. Interestingly, the median PFS of 22.9‐ and the 44.5‐month median OS observed in the present analysis appear to be superimposable to the data reported in the prospective trial.^8^ However, a 16.3 and 15.8‐month median PFS was documented in two prospective trials of idelalisib and anti‐CD20 monoclonal antibodies,^9^, ^19^ suggesting that patients' characteristics and different interpretation of imaging assessments may influence PFS across trials. Furthermore, the PFS value reported in this observational study should be interpreted with the notion that the timing of clinical assessment during follow‐up and that the modalities of defining progression are likely to be heterogeneous in the clinical practice, leading to possible overestimate of time to progression.

That being said, it is worth noting that PS ≥ 2 and as well ≥3 previous lines of therapy were associated with a shorter OS and PFS in our analysis and the a trend for shorter PFS was noted when comparing centers that included <5 patients and centers with ≥5 patients treated with IR. Our data also confirm that IR was not associated with an inferior PFS and OS in patients carrying a *TP53* disruption. Because the unmutated *IGHV* configuration was associated with shorter PFS at univariate analysis in our study and with a significantly shorter OS in the randomized study comparing IR versus placebo plus rituximab,^8^ further studies are required to conclusively establish the prognostic power of this immune‐genetic feature in patients treated with IR.

A valuable observation in our analysis is represented by the relatively high percentage of patients on treatment at 12 months (48%). Interestingly, we observed a strong independent influence of the expertise with IR on this objective parameter, with centers which treated at least five patients reporting a higher percentage of patients on treatment at 12 months (55.8% vs. 31.1%). This difference did not translate into a shorter OS, probably because of the efficacy of subsequent lines of therapy. The importance of specific expertise in the treatment of CLL has been previously recognized^20^ and our data reinforce this notion in R/R CLL patients treated with idelalisib. This finding is at variance with a large real‐world experience with ibrutinib, where despite variations in practice across centers, clear differences by the size, and type of the centers could not be clearly demonstrated.^21^

Earlier treatment discontinuation occurred in patients with ECOG PS ≥2 and with three or more previous lines of treatment. Furthermore, 24.3% and 11.8% of patients were still receiving the study drug at 24 and 36 months, respectively, in line with a previous observation in a small series of patients reporting 22% of patients on treatment at 24 months.^22^

The number of medical comorbidities did not portend inferior outcomes in our study, thus confirming in a real‐world setting the findings of a previous report of patients treated with idelalisib in clinical trials.^23^

The incidence of grade 3–4 infections and pneumonia (29.7% and 17%, respectively), diarrhea and colitis (27.1%), increased transaminase levels (5.9%) is similar to that observed in trials^8^, ^19^ and in a report describing the outcome in 68 patients treated in the UK and Ireland.^22^ The relatively low incidence of neutropenia (33.1%) may reflect the policy not to perform routine blood counts in the clinical practice at many centers.

The long follow‐up of our study allowed to document a 49.2% ORR in 59 patients who received a subsequent treatment, with a higher probability of response in patients who had received ≤2 lines of treatment prior to IR and with an encouraging median OS in patients who discontinued due to toxicity.

In conclusion, we hereby report the largest real‐life experience with IR in patients with R/R CLL treated and followed for a prolonged period of time at many centers across our country. This study shows (i) the overall efficacy of the IR combination, with a significant better outcome in terms of treatment duration at experienced centers; (ii) that the outcome was superimposable to the data previously reported in the randomized clinical trial; (iv) that despite therapy breaks in virtually all patients and frequent dose reductions, the percentage of patients on therapy over time was encouraging, especially in experienced centers, suggesting that toxicity with this drug may be manageable according to published recommendations^10^, ^11^, ^24^; (vi) that subsequent treatment was more efficacious after IR discontinuation due to adverse events.

Despite impressive progress with the recent approval of BTK and/or BCL2 inhibitors, the treatment of R/R CLL may represent an unmet need for patients carrying coexisting medical conditions which complicate these treatment options. Given the effectiveness of targeting the PI3K pathway,^24^ our analysis supports the notion that IR is a potentially efficacious regimen in the day‐to‐day practice, particularly so in experienced centers.

## CONFLICT OF INTEREST

Gian Matteo Rigolin, lecturing for Abbvie, Gilead and research funding from Gilead. Francesco Cavazzini, advisory board for Novartis; travel expenses from Janssen. Andrea Visentin, speaker's bureau from Janssen, Abbvie, Italfarmaco, Gilead, Gianluigi Reda, honoraria from AbbVie, Gilead, Janssen. Marta Coscia, honoraria from Janssen, Gilead, Abbvie, Shire; research support from Janssen and Karyopharm Therapeutics, Lucia Farina, advisory board for Janssen; lecturing for Abbvie. Gianluca Gaidano, advisory board and speaker's bureau for Janssen and Abbvie; Advisory Board for AstraZeneca. Marzia Varettoni, advisory board for Janssen, Roche, AstraZeneca. Travel expenses from Abbvie. Francesca M. Quaglia, advisory board for AstraZeneca; speaker bureau for Janssen. Luca Laurenti, advisory board and lecturing for Janssen, Gilead, Abbvie, Roche, and AstraZeneca. Roberto Marasca, research grant personal fees and nonfinancial support from Janssen, personal fees, and nonfinancial support from Gilead and personal fee from Abvie, Roche, Shire, Pfizer. Marco Montillo, honoraria from Abbvie, Gilead, Janssen, Roche; advisory board for Abbvie, Acerta/AstraZeneca, Gilead, Janssen, Roche, and Verastem, Robin Foà, Editorial boards and/or speaker's bureau for Janssen, AbbVie, Amgen, Novartis, Roche, Pfizer, Antonio Cuneo, Advisory board, and speaker bureau for Abbvie, Gilead, Janssen, AstraZeneca. No conflict of interest for all other authors.

## AUTHOR CONTRIBUTIONS

Antonio Cuneo, Robin Foà, Massimo Gentile, and Marco Montillo designed the research study. Gian Matteo Rigolin, Francesco Cavazzini, and Antonio Cuneo wrote the manuscript. Alfonso Piciocchi, Valentina Arena, Gian Matteo Rigolin, Francesco Cavazzini, Marco Vignetti, Antonio Cuneo, and Robin Foà analyzed and interpreted the data. Andrea Visentin, Gianluigi Reda, Giulia Zamprogna, Francesco Cavazzini, Francesca Cibien, Orsola Vitagliano, Marta Coscia, Lucia Farina, Gianluca Gaidano, Roberta Murru, Marzia Varettoni, Rossella Paolini, Paolo Sportoletti, Daniela Pietrasanta, Anna Lia Molinari, Francesca M. Quaglia, Luca Laurenti, Roberto Marasca, Monia Marchetti, Francesca R. Mauro, and Massimo Gentile contributed to the acquisition of data and interpreted the data. All authors substantially contributed to the acquisition of data revised, critically the manuscript, and approved the submitted and final version.

### PEER REVIEW

The peer review history for this article is available at https://publons.com/publon/10.1002/hon.2861.

## Supporting information

Supplementary Material 1

## Data Availability

The data that support the findings of this study are available from the corresponding author upon reasonable request.
