# Supplementary material for: Efficacy of idelalisib and rituximab in relapsed/refractory chronic lymphocytic leukemia treated outside of clinical trials. A report of the Gimema Working Group
Source: Hematol Oncol. 2021 Mar 26;39(3):326–35. doi: 10.1002/hon.2861 (PMC8451799; doi:10.1002/hon.2861)
Supplement: Supplementary file 1 — Supplementary Material 1 [file HON-39-326-s001.docx]

Supplementary Table 1. List of centers

| CENTRE | N pts |
| --- | --- |
| Haematology and Clinical Immunology Unit, Department of Medicine, University of Padua | 22 |
| Ematologia, Fondazione IRCCS Ca' Granda Ospedale Maggiore Policlinico di Milano | 12 |
| Haematology, Niguarda Cancer Centre, ASST Grande Ospedale Metropolitano Niguarda, Milan | 10 |
| Haematology, Ospedale Ca' Foncello, Treviso | 11 |
| Haematology, Cardarelli Hospital, Naples, | 9 |
| Department of Molecular Biotechnology and Health Sciences, University of Torino and Division of Haematology, A.O.U. Città della Salute e della Scienza di Torino | 8 |
| Division of Haematology , Fondazione IRCCS Istituto Nazionale dei Tumori, Milano | 6 |
| Division of Haematology, Department of Translational Medicine, Università del Piemonte Orientale, Novara | 6 |
| Haematology and Stem Cell Transplantation Unit, Ospedale Oncologico A. Businco, ARNAS "G. Brotzu", Cagliari | 5 |
| Division of Haematology, Fondazione IRCCS Policlinico San Matteo, Pavia | 5 |
| Haematology section, Department of Medical Sciences, University of Ferrara | 5 |
| Haematology Unit, Rovigo General Hospital U.O. di Ematologia – Ospedale di Rovigo | 5 |
| Department of Medicine and Surgery, Institute of Haematology and Centre for Hemato-Oncological Research, Ospedale S. Maria della Misericordia, Perugia | 4 |
| Haematology Division, Dipartimento Internistico Struttura Complessa di Ematologia Ospedale civile SS Antonio e Biagio, Alessandria | 4 |
| Haematology, Ospedale degli Infermi, Rimini, Italy; | 4 |
| Department of Medicine, Section of Haematology, University of Verona, Verona | 4 |
| Fondazione Policlinico Universitario A Gemelli. Roma | 3 |
| Department of Medical and Surgical Sciences, Section of Haematology, University of Modena and Reggio Emilia, Modena; | 3 |
| Oncology Unit, Cardinal Massaia Hospital, Asti, Italy. | 3 |
| Haematology Unit, Azienda Universitaria Ospedaliera Policlinico Vittorio Emanuele, Catania | 2 |
| Haematology, Department of Translational and Precision Medicine, 'Sapienza' University, Rome | 2 |
| Haematology, San Bortolo Hospital, Vicenza | 2 |
| Haematology Unit, A.O. Ospedali Riuniti Papardo-Piemonte, Messina | 2 |
| Haematology Unit Ospedale dell’Angelo, Mestre | 2 |
| Haematology and Stem Cell Transplant, Università Politecnica delle Marche, Ancona | 1 |
| Department of Haematology & Oncology, Azienda Ospedaliera Pugliese-Ciaccio, Catanzaro | 1 |
| Haematology, Federico II Università, Napoli | 1 |
| Haematology Unit, Ospedale V. Cervello, Palermo | 1 |
| Haematology, Azienda USL-IRCCS, Reggio Emilia | 1 |
| Haematology Unit Azienda Ospedaliera Universitaria Senese University of Siena | 1 |
| Haematology Unit, Hospital "A.Tortora," Salerno | 1 |
| Haematology and Cellular Therapy, "Ospedale C. e G. Mazzoni", Ascoli Piceno | 1 |
| Istituto Scientifico Romagnolo per lo Studio e la Cura dei Tumori (IRST) IRCCS, Meldola | 1 |
| Haematology Unit and Transplant Centre, Guglielmo da Saliceto Hospital, Piacenza | 1 |
| Total | 149 |

Supplementary Table 2. Principal clinical and biologic characteristics by ORR.

| Variable | No response  n=41 (27.5%) | CR + PR  n=108 (72.5%) | p |
| --- | --- | --- | --- |
| Age [median (range] years | 74.9 (46.8-90.8) | 69.9 (46.0-86.3)) | 0.01 |
| Age ≤70/>70 years | 12 (29.3) / 29 (70.7) | 54 (50.0) / 54 (50.0) | 0.04 |
| Gender M/F | 32 (78.0) / 9 (22.0) | 68 (63.0) / 40 (37.0) | 0.12 |
| ECOG PS (%) 0-1/≥2 | 30 (86.9) / 9 (23.1) | 95 (93.1) / 7 (6.9) | 0.02 |
| Comorbidities 0-1/≥2 | 11 (45.8) / 13 (54.2) | 32 (43.2) / 42 (56.8) | 1.00 |
| Creatinine clearance (ml/min) ≤70/>70 | 16 (51.6) / 15 (48.4) | 49 (55.7) / 39 (44.3) | 0.86 |
| Stage Rai III/IV or Binet C no/yes | 16 (39.0) / 25 (61.0) | 61 (56.5) / 47 (43.5) | 0.09 |
| Bulky lymph nodes (>5cm) no/yes | 19 (79.2) / 5 (20.8) | 72 (82.8) /15 (17.2) | 0.92 |
| *TP53* disruption yes/no* | 11 (26.8) / 30 (73.2) | 41(38.0)/ 67 (62.0) | 0.28 |
| *IGHV* Mutated/Unmutated | 6 (28.6) / 15 (71.4 | 22 (27.8) / 57 (72.2) | 1.00 |
| Previous lines of therapy <3/≥3 | 19 (46.3) / 22 (53.7) | 63 (58.3) / 45 (41.7) | 0.20 |

Legend: F=female; M=male.

*Del17p and/or *TP53* mut

Supplementary Table 3. Factors associated with R-Idelalisib treatment time ≥12 vs <12 months

| Variable | <12 months  n=77 (%) | >= 12 months  n= 72 (%) | p |
| --- | --- | --- | --- |
| Age [median (range] years | 69.6 (46.0-90.8) | 72.3 (48.0-85.1) | 0.14 |
| age ≤ 70 / > 70 years | 39 (50.6) / 38 (49.4) | 27 (37.5) / 45 (62.5) | 0.15 |
| Gender M/F | 54 (70.1) / 23 (29.9) | 46 (63.9) / 26 (36.1) | 0.52 |
| ECOG PS (%) ≥2 vs 0-1 | 16 (22.9) / 54 (77.1) | 0 (0.0) / 71 (100.0) | <0.001 |
| Comorbidities 0-1/≥2 | 24 (54.5) / 20 (45.5) | 19 (35.2) / 35 (64.8) | 0.09 |
| Clear Creatinine (ml/min) ≤ 70/>70 | 33 (55.0) / 27 (45.0) | 32 (54.2) / 27 (45.8) | 1.00 |
| Stage Rai III/IV or Binet C no/yes | 33 (42.9) / 44 (57.1) | 44 (61.1) / 28 (38.9) | 0.04 |
| Bulky lymph nodes (>5cm) no/yes | 46 (82.1) / 10 (17.9) | 45 (81.8) / 10 (18.2) | 1.00 |
| *TP53* disruption yes/no | 21 (27.3) / 56 (72.7) | 31 (43.1) / 41 (56.9) | 0.07 |
| *IGHV* Mutated/Unmutated | 14 (29.2) / 34 (70.8) | 14 (26.9) / 38 (73.1) | 0.98 |
| previous lines of therapy <3 /≥3 | 37 (48.1) / 40 (51.9) | 45 (62.5) / 27 (37.5) | 0.11 |
| N. of patients per centre ≥5 vs <5 | 46 (59.7) / 31 (40.3) | 58 (80.6) / 14 (19.4) | 0.007 |

Supplementary Table 4. Adverse events grade ≥3 (data available in 117 patients).

|  | PT | Grade | | |  |
| --- | --- | --- | --- | --- | --- |
|  |  | 3 | 4 | 5 | Total |
| Blood and lymphatic system disorders | Neutropenia | 22 | 17 | - | 39 |
|  | Thrombocytopenia | 1 | 1 | - | 2 |
|  | Anemia | 1 | - | - | 1 |
|  | Total | 24 | 18 | - | 42 |
| Infections | Pneumonia | 17 | - | - | 17 |
|  | Bronchitis | 6 | - | - | 6 |
|  | Cytomegalovirus | 3 | - | - | 3 |
|  | Sepsis | 2 | 1 | - | 3 |
|  | Sinusitis | 3 |  | - | 3 |
|  | Others | 2 | 1 | - | 3 |
|  | TYotal | 33 | 2 | - | 35 |
| Gastrointestinal disorders | Colitis | 10 |  | - | 10 |
|  | Diarrhoea | 20 | 1 | - | 21 |
|  | Gastritis | 1 |  | - | 1 |
|  | Total | 31 | 1 | - | 32 |
| Hepatobiliary disorders | Acute hepatitis | 1 |  | - | 1 |
|  | Transaminitis | 5 | 2 | - | 7 |
|  | Total | 6 | 2 | - | 8 |
| Skin disorders | Rash/Erythema | 8 | - |  | 8 |
|  | Total | 8 | - | - | 8 |
| Respiratory thoracic and mediastinal disorders | Pneumonitis | 2 | - | 1 | 3 |
|  | Cough | 2 | - | - | 2 |
|  | Dyspnoea/Respiratory failure | 1 | 1 | - | 2 |
|  | Total | 5 | 1 | 1 | 7 |
| Others | Infusion reaction | 1 | - | - | 1 |
|  | Leukoencephalopathy | - | - | 1 | 1 |
|  | Total | 1 | - | 1 | 2 |
| Overall total |  | 108 | 24 | 2 | 134 |
